# Efficacy and Safety of Etomidate Versus Ketamine for Endotracheal Intubation in Critically Ill Patients: A Systematic Review and Meta-Analysis

**DOI:** 10.7759/cureus.116406

**Published:** 2026-09-17

**Authors:** Zeeshan Umar, Tayyamn Tahseen, Aalyan Shafi, Samreen Gul, Banin Zainab, Amna Muhammad younus, Muhammad Awais Khan, Danesh Gul, Manqoosh Fatima, Zainab Razia, Abdul Bari

**Affiliations:** 1 Emergency Medicine, Pak Emirates Military Hospital (PEMH), Rawalpindi, PAK; 2 Cardiac Anesthesia, Rehmatul Lil Alameen Institute of Cardiology, Lahore, PAK; 3 Anaesthesiology, Karachi Institute of Medical Sciences (KIMS), Rawalpindi, PAK; 4 Anesthesia, Rehmatul Lil Alameen Institute of Cardiology, Lahore, PAK; 5 Emergency Medicine, Pakistan Naval Ship (PNS) Rahat Hospital, Karachi, PAK; 6 Anesthesiology, Mohtarma Benazir Bhutto Shaheed Medical College, Karachi, PAK; 7 Anesthesia, Koohi Goth Women’s Hospital, Karachi, PAK; 8 Emergency Medicine, Jinnah Postgraduate Medical Center, Karachi, PAK; 9 Anaesthesiology, Combined Military Hospital, Muzaffarabad, PAK; 10 Internal Medicine, Sindh Institute of Urology and Transplantation, Karachi, PAK; 11 Internal Medicine, Doctor Faisal Masood Teaching Hospital, Sargodha, PAK

**Keywords:** critically ill patients, emergency airway management, endotracheal intubation, etomidate, ketamine, meta-analysis, rapid sequence intubation

## Abstract

Rapid sequence intubation (RSI) is a commonly used technique for critically ill patients requiring emergency intubation, and the choice of induction agent is crucial for minimizing peri-intubation morbidity and maintaining hemodynamic stability. The most commonly used induction agents are etomidate and ketamine, although controversy remains about their effectiveness and safety. Therefore, this systematic review and meta-analysis compared the efficacy and safety of etomidate versus ketamine for endotracheal intubation in critically ill patients. Randomized controlled trials (RCTs) published between 2000 and May 2026 were systematically searched in PubMed, The Cochrane Library, and ScienceDirect. The included studies compared etomidate with ketamine for emergency endotracheal intubation in critically ill adults. We analyzed data with RevMan 5.4 and Mantel-Haenszel random-effects models and calculated risk ratios (RRs) with 95% confidence intervals (CIs). Seven RCTs involving 5234 patients were included in the final analysis. No significant difference was observed between etomidate and ketamine regarding 28-day mortality (RR 1.01; 95% confidence interval (CI) 0.93-1.10; p=0.84), cardiac arrest (RR 0.91; 95% CI 0.62-1.34; p = 0.64), failed intubation (RR 1.09; 95% CI 0.68-1.76; p=0.71), or vasopressor requirement (RR 1.27; 95% CI 0.94-1.72; p=0.13). Sensitivity analyses suggested possible hemodynamic differences between the two agents, which should be interpreted cautiously, and etomidate was relatively cardiovascularly stable in some analyses. Overall, etomidate and ketamine showed similar efficacy and safety for endotracheal intubation in critically ill patients. No statistically significant differences were observed between the two agents in mortality, cardiac arrest, failed intubation, or vasopressor use. Thus, clinicians should choose the induction agent on a case-by-case basis, based on patient, hemodynamic, and clinician-specific factors. Randomized trials in selected critically ill patient populations should be conducted on a larger scale.

## Introduction and background

Critically ill patients frequently require endotracheal intubation for airway protection and respiratory support in emergency departments and intensive care units [1]. Rapid sequence intubation (RSI) is commonly performed to provide rapid airway control in critically ill patients. Still, it is associated with many peri-intubation complications such as hypotension, hypoxemia, cardiovascular collapse, and cardiac arrest [2,3]. Therefore, the type of induction agent is essential for minimizing peri-intubation complications and maintaining hemodynamic stability [4]. Common induction agents include etomidate and ketamine, often selected for rapid sequence intubation because of their relatively favorable hemodynamic effects and their use in critically ill patients [4,5].

Etomidate has long been the agent of choice for inducing anesthesia in hemodynamically unstable patients, owing to its minimal cardiovascular depression and ability to preserve blood pressure [6]. But because of transient adrenal suppression and the possibility of affecting outcomes in the critically ill, questions have been raised about its safety [7]. Ketamine, by contrast, has sympathomimetic and bronchodilatory effects that can be beneficial during intubation in patients with hemodynamic instability or bronchospasm [8,9]. However, in patients with septic shock, endogenous catecholamine release may already be maximal, potentially attenuating the sympathomimetic effects of ketamine and allowing its intrinsic cardiovascular depressant effects to predominate. Consequently, ketamine may cause hypotension and, in severe cases, contribute to cardiovascular collapse [10]. Several studies have compared etomidate and ketamine for endotracheal intubation, yielding conflicting results regarding peri-intubation mortality and complications [11,12].

While several studies and meta-analyses have examined the comparative effectiveness of these induction agents, the evidence has been inconsistent, and newer studies have emerged, necessitating a new synthesis of the literature. Thus, this systematic review and meta-analysis aimed to compare the efficacy and safety of etomidate versus ketamine for endotracheal intubation in critically ill patients. The primary objective was to compare 28-day mortality between critically ill patients undergoing emergency endotracheal intubation with etomidate versus ketamine. Secondary objectives were to compare rates of failed intubation, cardiac arrest, and need for vasopressor support between the two induction agents.

## Review

We conducted this meta-analysis to compare ketamine with etomidate for endotracheal intubation in critically ill patients. This review was conducted in accordance with the Preferred Items for Systematic Reviews and Meta-analyses (PRISMA) guidelines, and the protocol was registered on PROSPERO (CRD420261384591) [13].

Search strategy

We used three main databases, including PubMed, Cochrane, and ScienceDirect, to search for articles relevant to our study. We used keywords combined with the Boolean operators AND and OR. The search strategy for PubMed was '(((ketamine) AND (etomidate)) AND (endotracheal intubation)) OR (emergency endotracheal intubation)' and the search strategy for ScienceDirect was '((ketamine) AND (etomidate) AND ((endotracheal intubation) OR (emergency endotracheal intubation)))’. For Cochrane, the search strategy looked like '(ketamine): ti, ab,kw AND (etomidate): ti, ab,kw AND (endotracheal intubation): ti, ab,kw OR (emergency endotracheal intubation): ti, ab,kw'. We searched for articles from 2000 to May 2026. For PubMed, we used the randomized controlled trials filter; for Science Direct, we used the research article and trials filters on Cochrane. We also searched the reference lists of the included articles for any relevant articles.

Eligibility criteria

Our review used specified eligibility criteria to screen articles for inclusion. The inclusion criteria consisted of studies with critically ill participants over 18 requiring emergency endotracheal intubation with ketamine and etomidate as interventions and comparators. Studies were included if they were randomized controlled trials (RCTs) published in English after 2000.

Similarly, studies were excluded if participants had contraindications to etomidate or ketamine, cardiac arrest, known pregnancy, or were not directly comparing etomidate and ketamine. Studies were excluded if they were cohort studies, case reports, cross-sectional studies, reviews, scientific abstracts, or unpublished trials.

Screening, selection, and data extraction

All articles retrieved using our search strategies across various databases were screened. Initially, duplicates were removed using the EndNote duplicate checker. The remaining articles underwent title and abstract screening, during which we removed all irrelevant articles. The remaining articles were searched for full text, and those with full-text availability were screened against our eligibility criteria for inclusion. External help was sought where required during the review. The whole process was carried out independently by two reviewers, and any dispute between the reviewers regarding the inclusion of an article was resolved with the help of a third reviewer.

Data extraction was carried out independently by one reviewer and reviewed by another author. The data extracted included study ID, sample size, study location, study design, Sample age, gender ratio, study objectives, baseline characteristics, details of interventions and comparators (including dosage and duration), and outcome data.

Quality assessment

Cochrane's risk-of-bias assessment tool for trials (Risk of Bias 2 (ROB2), Cochrane Collaboration, Copenhagen, Denmark) was used to assess the quality of the included RCTs. The tool consists of five domains and one overall domain. A study was classified as high risk if any domain was high risk and at least two domains had some concerns. A study with overall concerns had one domain with concerns; the remaining domains were low risk. Low risk was assigned to studies in which all domains were low risk of bias [14].

Data synthesis

We used Review Manager (RevMan), Version 5.4 (The Cochrane Collaboration, Copenhagen, Denmark; 2020) [15] to analyze the pooled and extracted data. For dichotomous variables, we used risk ratios as the outcome measure. The Mantel-Haenszel random-effects model was used throughout the study for all dichotomous variables. A 95% confidence interval was used, and statistical significance was considered if the p-value was below 0.05. I2 was the measure of statistical heterogeneity, with I2>40% considered significant. I2<25 was considered low, 26-50 was considered mild, 51-75 was considered moderate, and >75% was considered high. Forest plots were used to present the results for all the outcomes. Subgroup analysis and funnel plots to assess publication bias could not be conducted due to limited data availability and a limited number of trials, respectively. A leave-one-out sensitivity analysis was conducted to rule out potential sources of heterogeneity.

Results

We initially identified 2574 articles from our three databases using the above-mentioned keywords. The articles were imported into EndNote to remove duplicates (78). Out of the remaining 2496 articles, 2423 were deemed irrelevant. The full text of five articles could not be found, and, finally, selection criteria were applied to the 68 remaining articles, of which 61 were removed for reasons listed in the flowchart. There was confusion about whether to include unpublished trials, as the previous meta-analysis had included them, but we decided not to. This review consisted of seven published RCTs [12,16-21]. The entire screening process is summarized in the PRISMA flowchart in Figure 1.

**Figure 1:**
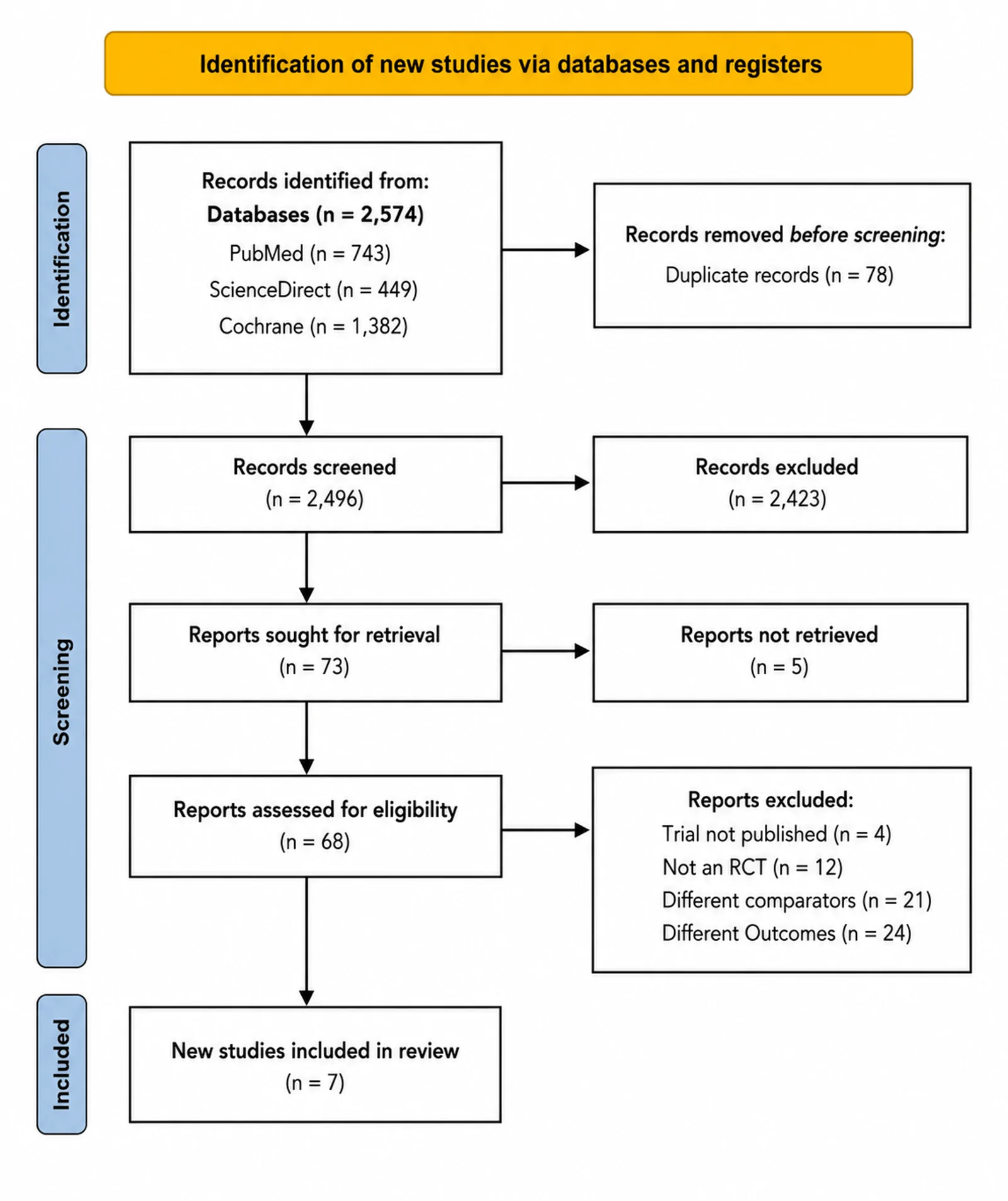
PRISMA flowchart for study screening and selection PRISMA: Preferred Reporting Items for Systematic Reviews and Meta-Analyses.

Characteristics of Studies

We included seven trials [12,16-20] in the final analysis. Three of them were conducted in the United States, one each in India, France, the Netherlands, and Thailand. Two studies reported age as the median, while five trials reported age as the mean. This review consisted of 5234 participants, of whom 2000 (38.2%) were women. In the studies, 0.2-0.3 mg/kg was the common dosage for etomidate and 1-2 mg/kg for ketamine, while one used 0.5 mg/kg. In the review, 2676 (51.1%) participants received etomidate, while 2558 (48.9%) received ketamine for induction of anesthesia before endotracheal intubation. The characteristics have been summarized in Table 1.

**Table 1:** Characteristics of the included studies Data are presented as mean (standard deviation (SD)) or median (interquartile range (IQR)), as reported in the original studies. Sample size and female participants are presented as number (n). Age values in parentheses indicate the SD or IQR. Drug doses are presented as dose (mg/kg), and the values in parentheses indicate the number of participants (n) receiving that dose. ED=emergency department; ICU=intensive care unit.

Study	Location	Age	Sample size (number of participants)	Females (number of participants)	Etomidate dose (number of participants)	Ketamine dose (number of participants)
Agarwal D et al. 2025 [16]	India	Mean (SD) Ketamine: 70.5 (14.9); Etomidate: 73.2 (12.6)	80	33	0.2-0.3 mg/kg (40)	1-2 mg/kg (40)
Casey JD et al. 2025 [17]	USA (6 EDs and 8 ICUs)	Median (IQR) Ketamine: 60 (45-69); Etomidate: 60 (44–69)	2365	990	0.3 mg/kg (1189)	2.0 mg/kg (1176)
Jabre P et al. 2009 [18]	France (12 EDs and 65 ICUs)	Mean (SD) Ketamine: 59 (19); Etomidate: 57 (18)	469	189	0.3 mg/kg (234)	2.0 mg/kg (235)
Matchett G et al. 2022 [19]	USA	Mean (SD) Ketamine: 55.4 (16); Etomidate: 55.8 (15.2)	791	303	0.2-0.3 mg/kg (396)	1-2 mg/kg (395)
Punt C et al. 2014 [21]	Netherlands (ICU)	Mean (SD) Ketamine: 67 (13); Etomidate: 66 (14)	301	117	0.2-0.3 mg/kg (161)	0.5 mg/kg (140)
Srivilaithon W et al. 2023 [12]	Thailand (ED)	Mean (SD) Ketamine: 70.5 (14.9); Etomidate: 73.2 (12.6)	260	107	0.2-0.3 mg/kg (130)	1-2 mg/kg (130)
Upchurch CP et al. 2018 [20]	USA (ED)	Median (IQR) Ketamine: 37.1 (26.6-53.5); Etomidate: 39.8 (26-57)	968	261	0.3 mg/kg (526)	1-2 mg/kg (442)

Risk of Bias

All of the included trials were high quality, that is, low risk of bias, except the study by Agarwal et al. (2025) [16], which had a high risk of bias due to some concerns in domain 2: the trial was single-blinded, and there was potential for differences in various co-interventions between groups. Similarly, the risk of selective reporting could not be excluded for this trial as there was no trial registration number or published protocol. A summary of the risk of bias is provided in Figure 2.

**Figure 2:**
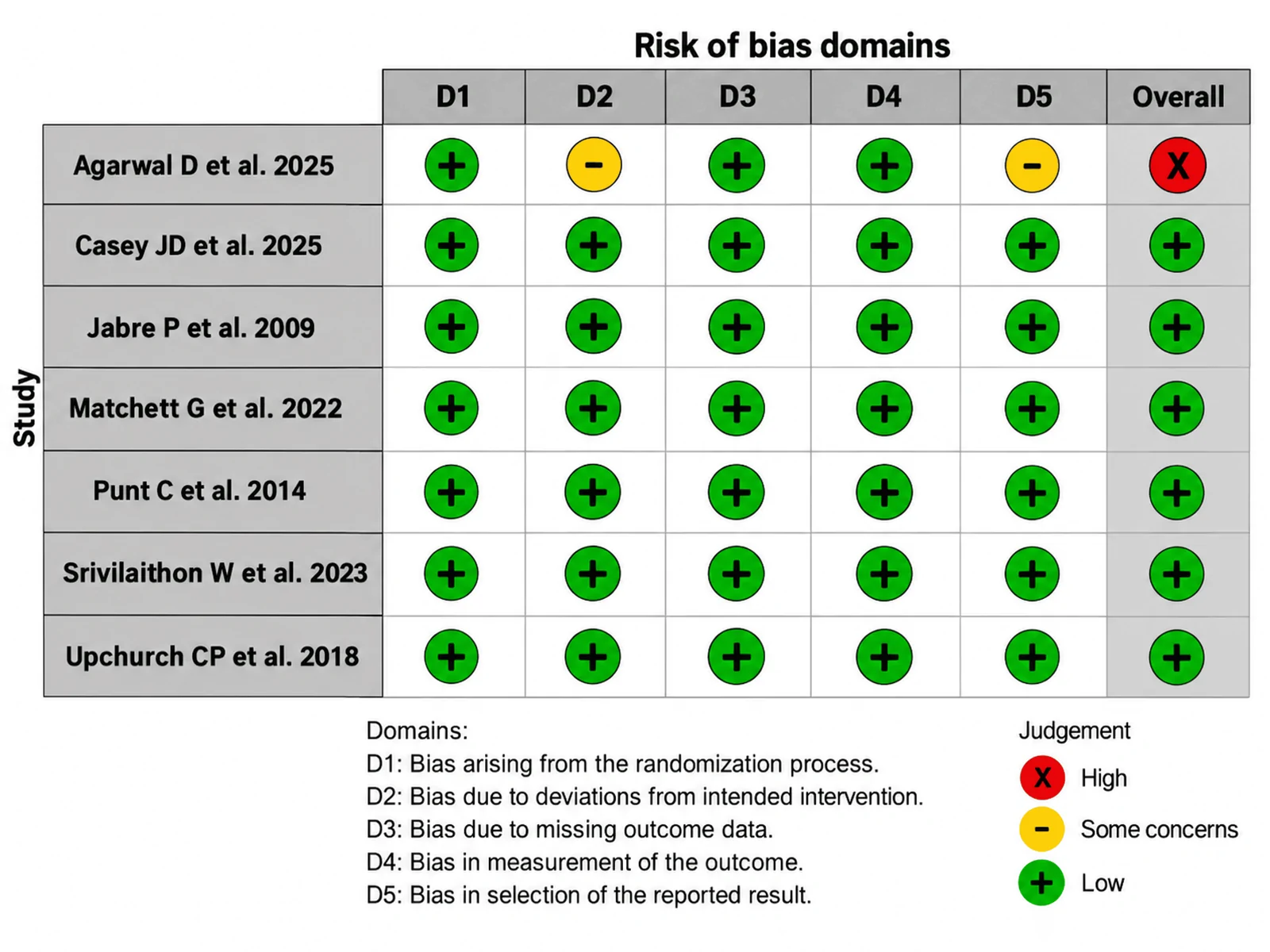
Summary of risk of bias Risk of bias assessment of the included studies using the Cochrane Risk of Bias 2 (RoB 2) tool. D1=Bias arising from the randomization process; D2=Bias due to deviations from intended interventions; D3=Bias due to missing outcome data; D4=Bias in measurement of the outcome; D5=Bias in selection of the reported result. Green (+) indicates low risk of bias, yellow (−) indicates some concerns, and red (×) indicates high risk of bias.

Mortality at 28 Days

Mortality was assessed at 28 days and reported by all included trials, with 753 events in the etomidate group and 723 in the ketamine group. The analysis showed no significant difference in mortality between the two groups, with no observed heterogeneity (RR 1.01; 95% CI 0.93-1.10; p=0.84). I2=0%. The forest plot for this is shown in Figure 3.

**Figure 3:**
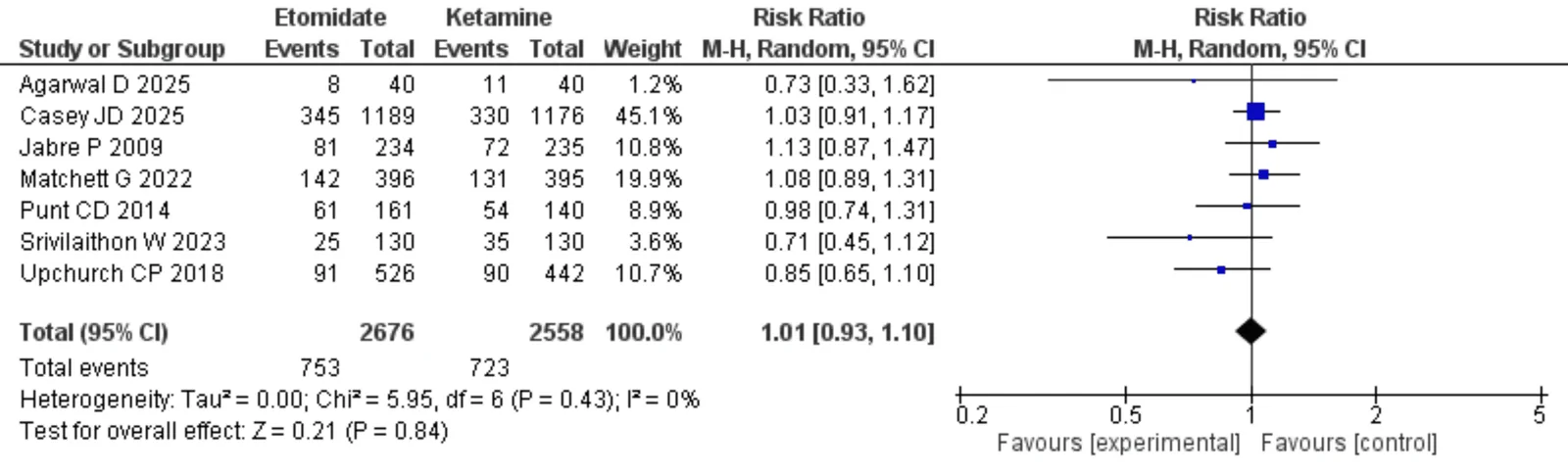
Forest plot for mortality

Need for Vasopressor Support

Although the numbers showed that the need for vasopressor support after induction differed between the two groups, the analysis showed no substantial difference in this outcome between etomidate and ketamine, and the observed heterogeneity was high, i.e., I2=90% (RR 1.27; 95% CI 0.94, 1.72, p=0.13). The forest plot for this outcome is given in Figure 4.

**Figure 4:**
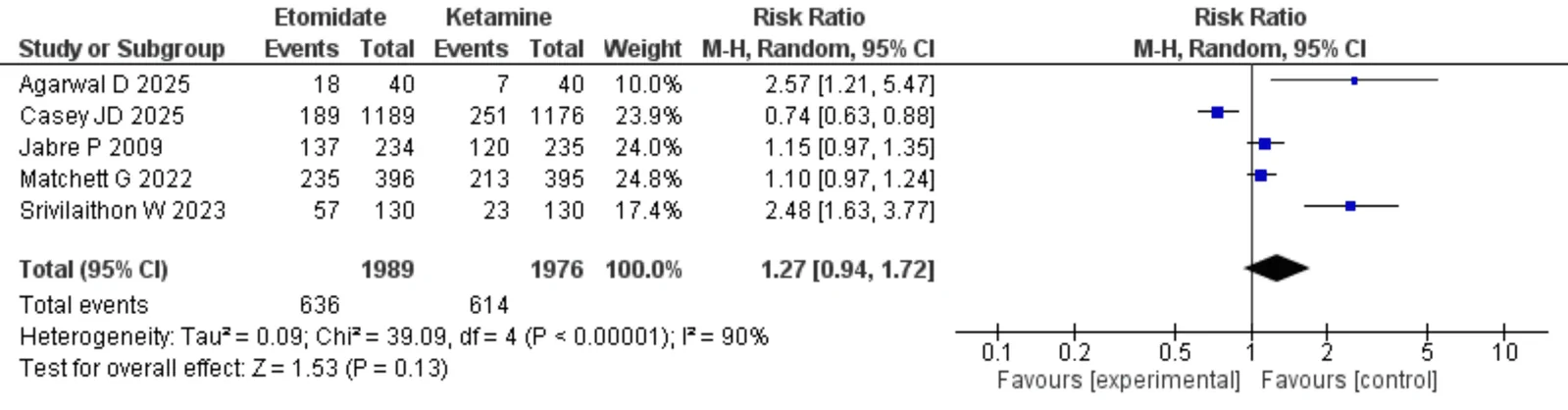
Forest plot for vasopressor support

Although leave-one-out sensitivity analysis did not reduce heterogeneity, the difference became significant in favor of ketamine after removing Casey et al. 2025 [17] (RR 1.47; 95% CI 1.08, 1.99; P=0.02; I2=84%).

Cardiac Arrest and Failed Intubation

The risk of cardiac arrest showed no significant difference between the etomidate and ketamine groups, and the observed heterogeneity was low (RR 0.91; 95% CI 0.62-1.34; p=0.64; I2=19%). The results are shown in Figure 5.

**Figure 5:**
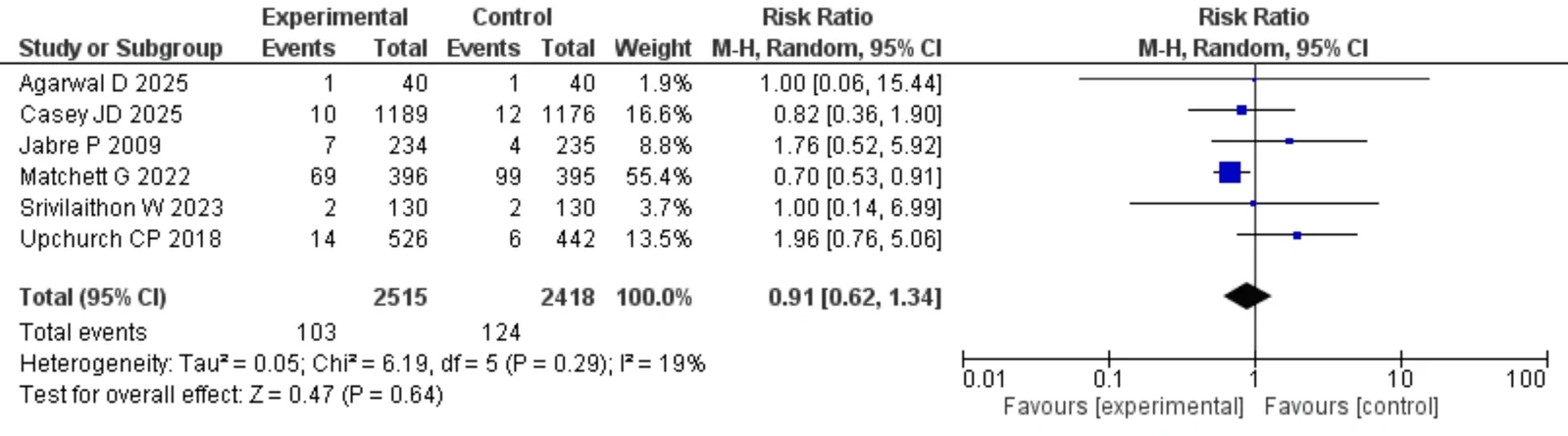
Forest plot for cardiac arrest

Sensitivity analysis showed a significant difference in the risk of cardiac arrest in favor of etomidate, with no heterogeneity after removal of Upchurch et al. 2018 [20] (RR 0.74; 95% CI 0.58, 0.95, p=0.02).

Similarly, no significant difference was observed between etomidate and ketamine for the failure-to-intubate outcome, and the statistical heterogeneity was zero (RR 1.09; 95% CI 0.68-1.76; p=0.71). The forest plot for failed intubations is shown in Figure 6.

**Figure 6:**
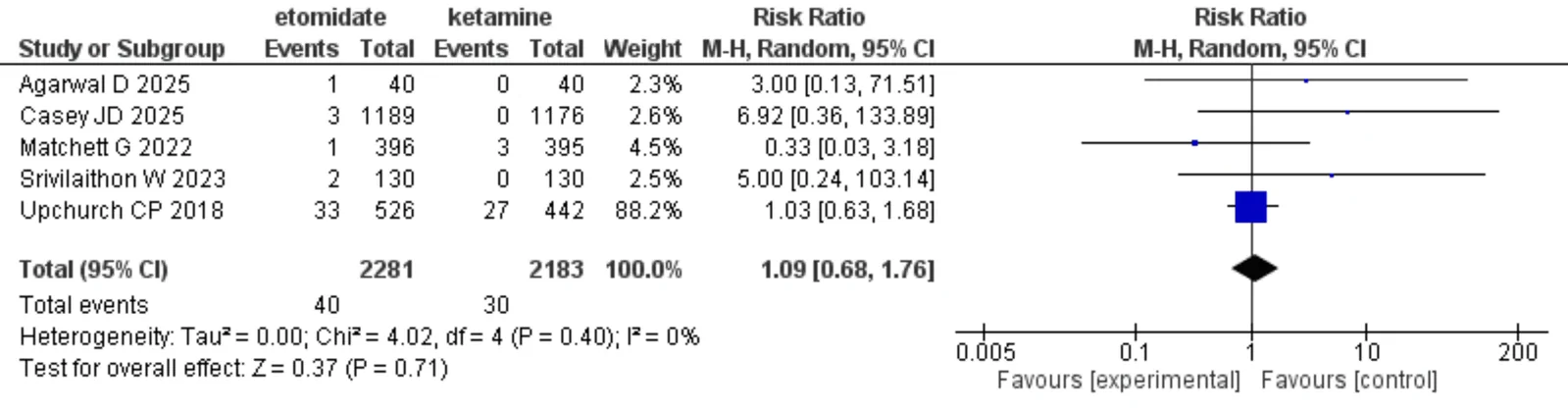
Forest plot for failed intubations

Discussion

In this systematic review and meta-analysis, seven randomized controlled trials involving 5234 patients were analyzed to compare the efficacy and safety of intubation with etomidate versus ketamine in critically ill patients. Overall, there was no difference between the 28-day mortality, risk of cardiac arrest, failed intubation, or requirement for vasopressor support between etomidate and ketamine. The findings are consistent with a recent meta-analysis that found no difference in 28-day mortality, cardiac arrest, or intubation success between etomidate and ketamine in critically ill patients intubated with rapid-sequence induction [22]. The results of this study indicate similar overall clinical effects of the two induction agents in rapid-sequence intubation in critically ill adults.

The lack of any difference in mortality between the two agents is clinically relevant because adrenal suppression has been a concern with etomidate and has historically raised concerns about its use in critically ill and septic patients. Previously, studies and systematic reviews did not show any significant differences in short-term mortality after a single dose of etomidate [12,23]. Likewise, the sympathomimetic effect and the maintenance of airway reflexes and cardiovascular stability have been regarded as beneficial effects of ketamine [24]. These findings suggest that the potential adverse effects of either induction agent should be interpreted in the context of overall clinical outcomes rather than the pharmacologic properties of the induction agents alone. In critically ill patients, clinical outcomes are multifactorial and may be influenced by baseline hemodynamic status and the choice of induction agent, among other patient and procedural factors [25]. 

Similarly, this analysis failed to demonstrate any difference between the intubability of failed intubations with etomidate versus ketamine, indicating that both are equally effective for intubation success. This is consistent with previously published systematic reviews and meta-analyses that reported similar success rates for first-pass intubation with the two induction agents in rapid-sequence intubation [11]. Other variables that determine the success of intubation include operator experience, patient physiology, airway difficulty, and the timing of the intervention, which may be more important in the successful control of the airway in the critically ill patient than the type of induction agent used [1]. Thus, the similar intubation outcomes observed in this analysis suggest that neither ketamine nor etomidate provides a clear advantage in terms of airway control when used for induction. The choice of the agent may therefore be better guided by the patient's physiological condition and the anticipated hemodynamic response rather than by expectations of improved intubation success alone.

There was no significant difference in post-induction vasopressor use between ketamine and etomidate. However, the included studies did not provide sufficient data to determine whether patients already receiving vasopressors before induction experienced increased vasopressor requirements after administration of either induction agent. This finding is consistent with previous meta-analyses showing similar vasopressor administration during emergency intubation with ketamine and etomidate [26]. Interestingly, sensitivity analysis revealed that the use of vasopressors was significantly lower with ketamine after excluding the large study by Casey et al., which may indicate a benefit of ketamine in certain patient subsets for supporting hemodynamics. Ketamine is a sympathomimetic drug that may be useful for maintaining blood pressure during induction, particularly in septic and distributive shock patients. Such positive effects may be less pronounced in those who are very ill, as their catecholamine levels are depleted in these cases, which may explain the variations between studies. The substantial heterogeneity in vasopressor requirements reflects differences across the included critically ill populations and study protocols. Differences in baseline cardiovascular status, shock severity, pre-induction vasopressor exposure, induction dosing, and resuscitation practices likely contribute to this variability. Importantly, the available trials did not consistently provide enough information to determine whether patients already receiving vasopressors before induction required additional vasopressor support after ketamine or etomidate. Therefore, the pooled estimate should not be interpreted as demonstrating equivalent hemodynamic effects in all shock states.

The pooled analysis also showed no difference in cardiac arrest rates between the two groups, consistent with previous meta-analyses of cardiac arrest post-induction in critically ill patients, which found no difference in cardiac arrest incidence between ketamine and etomidate [22]. However, sensitivity analysis in the present study favored etomidate after exclusion of the Upchurch et al. study. This finding may be related to the relatively stable cardiovascular profile of etomidate induction. The rates for the events, however, are relatively low, and there is some variation across the different sensitivity analyses, suggesting that the true difference between the two agents in peri-intubation cardiac arrest remains unclear. Although the sensitivity analysis favored etomidate after excluding one study, this finding should be interpreted cautiously because cardiac arrest events were relatively infrequent and the result depended on excluding a single trial. This finding therefore does not establish a definitive cardiovascular safety advantage for etomidate, but it highlights the importance of considering baseline hemodynamic status when selecting an induction agent. Therefore, the choice between ketamine and etomidate must be individualized, based on the patient's characteristics, underlying hemodynamic status, and the clinician's preference.

From a clinical perspective, these findings support an individualized approach to induction-agent selection during emergency intubation. Although neither ketamine nor etomidate demonstrated clear superiority for mortality, failed intubation, cardiac arrest, or vasopressor use in the pooled analyses, the sensitivity analyses and substantial heterogeneity in hemodynamic outcomes suggest that treatment effects may differ across patient populations. In particular, patients with severe hemodynamic instability or septic shock may require careful consideration of their underlying cardiovascular and catecholamine status before induction. Thus, the choice of induction agent should be guided by patient physiology, anticipated hemodynamic response, and clinician judgment rather than by a universal preference for either agent.

This review has several strengths. Only randomized controlled trials were included to minimize confounding and selection bias. Moreover, the methodological quality of the studies included in the analysis was overall high, and the risk of bias was low. Further adding to the power of the analysis and the relevance of the findings, more recently published large multicenter trials were also included in the meta-analysis. However, certain limitations should also be acknowledged. Some secondary outcomes, such as use of vasopressors, showed significant differences. This heterogeneity could be due to differences in patient populations, intubation parameters, ketamine doses, outcome definitions, and/or other factors. Furthermore, the small amount of data available prevented subgroup analyses by severity of illness, trauma, and sepsis. The small number of studies included in the analysis also prevented a thorough analysis of publication bias.

## Conclusions

This systematic review and meta-analysis indicate that endotracheal intubation with etomidate and ketamine is equally effective and safe in critically ill patients. No significant differences between the two agents were found in mortality, failed intubation, cardiac arrest, or vasopressor use. The overall evidence failed to show a clear hemodynamic advantage of one agent over the other. However, the results of the sensitivity analyses were inconsistent and must be interpreted with caution. Therefore, the selection of induction agents must reflect the individual patient, hemodynamic status, and the clinician’s experience. Further RCTs involving larger cohorts of critically ill patients are needed to define the optimal induction agent better.
